# Editorial: CRISPR-aided bioengineering for value-added product development

**DOI:** 10.3389/fbioe.2023.1340377

**Published:** 2023-12-13

**Authors:** Anindya Bandyopadhyay, Michael Köpke, Tae Seok Moon

**Affiliations:** ^1^ International Maize and Wheat Improvement Center, Texcoco, Mexico; ^2^ LanzaTech Inc., Skokie, IL, United States; ^3^ Moonshot Bio Inc., North Andover, MA, United States

**Keywords:** CRISPR, gene editing, biotechnology, bioprospecting, computational design, CRISPRi, base editor, CRISPR methylation

Since its discovery and development, CRISPR technology has revolutionized biology with the possibility of editing the genome of organisms from any kingdom of life (Wang and Doudna, 2023). The technology allows for editing multiple targets in one shot, base editing at single nucleotide resolution, selectively activating or repressing gene expression, and modifying the epigenetic landscape, considered impossible or difficult to achieve previously. This wave of CRISPR technology has reached bioengineering and synthetic biology with the promise of rapid and multiplexed cellular engineering, generation of gene variants, screening for certain functions or traits, selective modulation of metabolism, and easy and fast removal of competitive pathways. A myriad of fields of biology, from the medical sector to agriculture and industrial biotechnology, are embracing this tool, leading to rapid development and discovery, and a vast CRISPR-based market is supposed to surpass 15.84 billion USD by 2028 (Bloomberg, 2022).Clinical applications, such as treating diseases or finding novel drug targets, gained momentum with the first government approval of CRISPR-based treatment for sickle-cell disease and β-thalassaemia in the UK (Wong, 2023), as did the development of new diagnostics tools. Industrial biotechnology and agriculture immensely benefit from this technology for the development and optimization of highly productive bacteria, yeasts, algae, filamentous fungi, and crops. With the advent of CRISPR tools and a decade-long development (Wang and Doudna, 2023), it’s a timely topic to discuss recent advancements. The nine articles in this Research Topic cover the latest progress in the development and application of CRISPR tools in sustainability and biomanufacturing, diagnostics and drug development, and fundamental tools.

The first category is the development and applications of CRISPR tools in metabolic engineering for sustainable chemical and biofuel production. For example, gene expression control in metabolic engineering is a critical challenge. Kim and Lee address this issue by developing and implementing CRISPR interference (CRISPRi) tools to repress target genes in a multiplexed way to enhance the biomanufacturing of isoprenol, a fragrance molecule and biofuel precursor. Specifically, the authors target 32 essential and nonessential genes in *E. coli*, enabling simultaneous gene repression and achieving up to a 4.5-fold increase in an isoprenol titer. Additionally, a 2L fed-batch fermentation leads to a record titer of 12.4 g/L, demonstrating the tool’s utility. Another challenge is that not all host platforms are as advanced as model organisms such as *E. coli*. CRISPR tools open up an opportunity to access such non-traditional hosts with unique properties such as the fixation of CO_2_ for bioengineering. Dhokane et al. review CRISPR-based bioengineering strategies and approaches for microalgae, which are attractive candidates for biomanufacturing due to their high photosynthetic efficiency. Promising recent progress has been made in the adaptation and use of CRISPR tools to several microalgae species, including *Chlamydomonas reinhardtii*, *Nannochloropsis* spp., *Chlorella* spp., and *Phaeodactylum tricornutum* for the production of chemicals, cosmetics, nutrition, and biofuels, but further optimization is still needed.

Acetogenic bacteria are another emerging platform for the conversion of CO_2_ and industrial waste streams into fuels, chemicals, proteins, and materials. Three articles describe the growing applications of different CRISPR tools for various industrially relevant acetogens. Nwaokorie et al. describe the use of CRISPR-Cas9 in *Clostridium autoethanogenum* to study genes involved in carbon fixation. Even though this organism is used at the commercial scale today, there are still gaps in our understanding of carbon fixation that CRISPR tools can help to unravel. By deleting two genes of unknown function associated with a known C1-fixation gene cluster, the authors were able to show a significant impact on growth, product, and proteome profiles, contributing to a better understanding of genotype-phenotype relationships. Seys et al. expanded on the CRISPR-Cas9 system in *C*. *autoethanogenum* by adding a base editor function. Base editors Target-AID and Target-AID-NG derived from the Cas9 nuclease were used to introduce nonsense mutations into four different coding sequences. The authors also demonstrated that the system can be multiplexed, but they also highlighted some important limitations of current base editors that need further research, including off-target mutations or mixed genotypes, and they suggested best practices that should be considered. In some acetogens such as *Acetobacterium woodii*, however, attempts to install the CRISPR-Cas9 system were unsuccessful. Poulalier-Delavelle et al. explored endogenous CRISPR-Cas systems in acetogens. They developed an algorithm to automate the identification of PAM candidate sequences. With the help of the algorithm, they were able to successfully hijack the endogenous Type I-B CRISPR/Cas system of *A. woodie* and create in-frame deletions. The devised workflow was also successfully applied to the Type I-B CRISPR/Cas system of *C*. *autoethanogenum*.

Beyond applications in metabolic engineering and sustainability, CRISPR-Cas9 systems also have numerous applications in the medical field and can accelerate drug discovery and diagnostics. Li et al. summarize advances in CRISPR technology for rapid diagnosis of pathogenic infections via Recombinase-aided amplification (RAA). RAA is an isothermal nucleic acid amplification technology that offers advantages such as simplicity, speed, precision, energy efficiency, and convenient operation and can be integrated with CRISPR technology, enabling more convenient and intuitive determination of detection results. This integration has significantly expanded the application of RAA in pathogen detection. The discovery of new pharmaceuticals, antibiotics, therapeutic proteins, vaccine adjuvants, and bioactive natural products is often hampered by the inability to genetically engineer natural organisms, and prototyping pathways in other systems can be challenging. Hu et al. developed a highly efficient CRISPR/Cas9-mediated genome editing system in a vancomycin-producing strain of *Amycolatopsis keratiniphila*. The system enables the deletion of large fragments (up to 87.5 kb), which was not possible previously, and the generation of mutants with up to a 40% increase in the vancomycin yield. Plant cell-culture-based biomanufacturing is rapidly becoming an effective approach for the production of high-value plant-derived products, but their biomanufacturing often suffers from limited metabolic flux if they are not directly derived from the core metabolism. Brzycki Newton et al. developed a strategy using CRISPR-guided DNA methylation and chemical inhibitors to control flux to target pathways. They demonstrated the effectiveness of this approach by optimizing the biosynthesis of the potent anticancer drug paclitaxel (Taxol) via the phenylpropanoid pathway in *Taxus chinensis*. To do this, they knocked down the expression of specific enzymes in metabolism using a CRISPR-guided plant DNA methyltransferase (NtDRM) and by chemical inhibition, leading to a 25-fold increase in paclitaxel accumulation.

Since CRISPR-Cas9 as a genome-editing technology was first described (Jinek et al., 2012), it has been applied in many innovative ways including the ones described above–CRISPR interference Kim and Lee, CRISPR multiplexing Kim and Lee; Seys et al., CRISPR base editing Seys et al., and CRISPR-guided methylation Brzycki Newton et al. Another interesting opportunity is the use of pooled CRISPR gRNA libraries to do genome-wide fitness screening. Fundamental to support all these tools are CRISPR design algorithms. Simirenko et al. describes a new open-access design tool for targeted and genome-scale gRNA design (“gRNA-SeqRET”). This *in silico* tool aids the automatic extraction of target regions and the construction of pooled gRNA assemblies, and it is universally applicable for any prokaryote or eukaryote.

This Research Topic of these nine articles is just a snapshot of the current development and myriad of applications of CRISPR tools. With more real-world applications of powerful CRISPR technologies, careful consideration of their safety and ethical consequences should be made (Moon, 2023). The biological research fields will continue to be disrupted by innovative applications of CRISPR.
